# Ligand- and Structure-Based Virtual Screening Identifies New Inhibitors of the Interaction of the SARS-CoV-2 Spike Protein with the ACE2 Host Receptor

**DOI:** 10.3390/pharmaceutics16050613

**Published:** 2024-05-01

**Authors:** Timoteo Delgado-Maldonado, Alonzo González-González, Adriana Moreno-Rodríguez, Virgilio Bocanegra-García, Ana Verónica Martinez-Vazquez, Erick de Jesús de Luna-Santillana, Gerard Pujadas, Guadalupe Rojas-Verde, Edgar E. Lara-Ramírez, Gildardo Rivera

**Affiliations:** 1 Laboratorio de Biotecnología Farmacéutica, Centro de Biotecnología Genómica, Instituto Politécnico Nacional, Reynosa 88710, Mexico; titi_999@live.com (T.D.-M.); al.gonzalez.gonzalez88@gmail.com (A.G.-G.); elarar0700@hotmail.com (E.E.L.-R.); 2 Laboratorio de Estudios Epidemiológicos, Clínicos, Diseños Experimentales e Investigación, Facultad de Ciencias Químicas, Universidad Autónoma “Benito Juárez” de Oaxaca, Avenida Universidad S/N, Ex Hacienda Cinco Señores, Oaxaca 68120, Mexico; arimor10@hotmail.com; 3 Centro de Biotecnología Genómica, Instituto Politécnico Nacional, Reynosa 88710, Mexico; vbocanegra@ipn.mx (V.B.-G.); avmartinez@gmail.com (A.V.M.-V.); edeluna@ipn.mx (E.d.J.d.L.-S.); 4 Departament de Bioquímica i Biotecnologia, Research Group in Cheminformatics & Nutrition, Campus de Sescelades, Universitat Rovira i Virgili, 43007 Tarragona, Spain; gerard.pujadas@gmail.com; 5 Instituto de Biotecnología, Facultad de Ciencias Biológicas, Universidad Autónoma de Nuevo León, Monterrey 66451, Mexico; grojasverde@gmail.com

**Keywords:** COVID-19, SARS-CoV-2, spike, protein–protein interaction inhibitors, inhibitors viral entry

## Abstract

The Severe Acute Respiratory Syndrome Coronavirus 2 (SARS-CoV-2) is a fast-spreading viral pathogen and poses a serious threat to human health. New SARS-CoV-2 variants have been arising worldwide; therefore, is necessary to explore more therapeutic options. The interaction of the viral spike (S) protein with the angiotensin-converting enzyme 2 (ACE2) host receptor is an attractive drug target to prevent the infection via the inhibition of virus cell entry. In this study, Ligand- and Structure-Based Virtual Screening (LBVS and SBVS) was performed to propose potential inhibitors capable of blocking the S receptor-binding domain (RBD) and ACE2 interaction. The best five lead compounds were confirmed as inhibitors through ELISA-based enzyme assays. The docking studies and molecular dynamic (MD) simulations of the selected compounds maintained the molecular interaction and stability (RMSD fluctuations less than 5 Å) with key residues of the S protein. The compounds DRI-1, DRI-2, DRI-3, DRI-4, and DRI-5 efficiently block the interaction between the SARS-CoV-2 spike protein and receptor ACE2 (from 69.90 to 99.65% of inhibition) at 50 µM. The most potent inhibitors were DRI-2 (IC_50_ = 8.8 µM) and DRI-3 (IC_50_ = 2.1 µM) and have an acceptable profile of cytotoxicity (CC_50_ > 90 µM). Therefore, these compounds could be good candidates for further SARS-CoV-2 preclinical experiments.

## 1. Introduction

The coronavirus disease (COVID-19) caused by the Severe Acute Respiratory Syn-drome Coronavirus 2 (SARS-CoV-2) produced more than 7 million deaths worldwide as of February 2024 [1,2]. SARS-CoV-2 is a highly pathogenic *Betacoronavirus* for humans, like the earlier SARS-CoV and MERS-CoV, which are also characterized as causing the life-threatening severe respiratory syndrome [3]. The SARS-CoV-2 genome has a length of size of 29.8–29.9 kb and encodes the envelope protein (E), membrane protein (M), nucleocapsid protein (N), and spike glycoprotein structural proteins, as well as the fifteen (1–15) non-structural proteins (Nsps), produced after the cleavage of the translated polypeptides open reading frames (ORF1a and ORF1b) by Papain-like Protease (PLPro) and Main protease (Mpro) [4,5,6]. Some of these viral proteins have been proposed as molecular targets in drug discovery to treat COVID-19 [7].

The S glycoprotein, involved in the early stages of infection, is one of the most studied and represents a suitable target in drug design [8,9]. The spike is anchored to the viral envelope by the transmembrane segment and decorates the surface of the virion with the large ectodomain [10]. Through the receptor-binding domain (RBD) region, the spike interacts with and binds to human angiotensin-converting enzyme 2 (ACE2). The main contact residues of the SARS-CoV-2 RBD–ACE2 are Lys417, Gly446, Tyr449, Tyr453, Leu455, Phe456, Ala475, Phe486, Asn487, Tyr489, Gln493, G496, Gln498, Thr500, Asn501, Gly502, and Tyr505 [11]. Based on this knowledge, RBD-specific monoclonal antibodies have been developed as potent blockers of the virus entry; however, this therapy may show problems, such as solubility, poor bioavailability via the oral route, and serious immunogenicity, creating the need of develop alternative drug therapies [12].

Computational aided approaches have helped to devise new SARS-CoV-2 treatments. For example, Yang et al. [13], based on in silico studies and in vitro assays, demonstrated that drug-approved itraconazole (IC_50_ = 0.45 µM) and estradiol benzoate (IC_50_ = 1.02 µM) block the S protein and inhibit the protein-mediated intercellular fusion in cell experiments. However, further studies indicated that the therapeutic use of itraconazole should be handled with caution and should be administered carefully due to its non-linear pharmacokinetics [14]. Meanwhile, estradiol benzoate showed significant toxicity in meiobenthic nematodes starting from the concentration of 4.3 ng/L [15]. Also, the exposure of prostate human cells to estradiol benzoate showed changes in the expression levels of Nanog homeobox (NANOG), C-C motif chemokine ligand 2 (CCL2), and bone morphogenetic protein receptor type 2 (BMPR2) genes [16].

**Figure 1:**
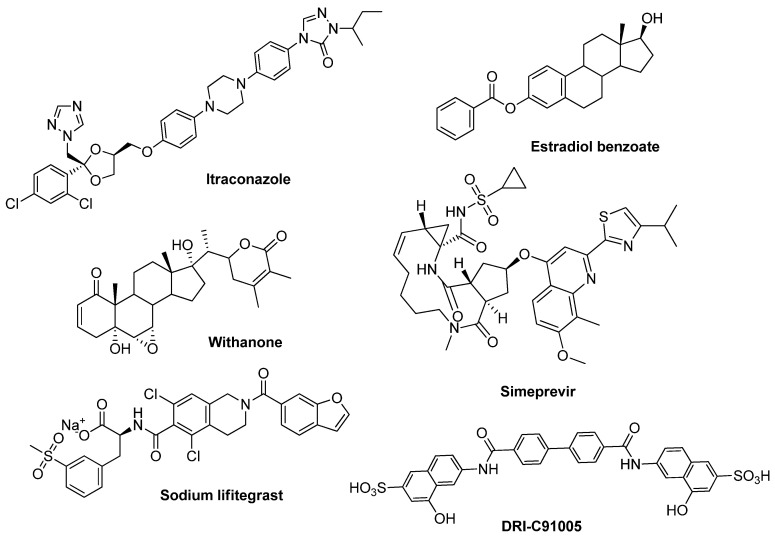
Chemical structure of spike protein inhibitors.

Moreover protein–protein interaction (PPI) inhibitors of the S protein and its host cell receptor ACE2 have been reported (Figure 1), including small molecules, like withanone (IC_50_ = 0.33 ng/mL), sodium lifitegrast (*K_D_* = 1.92 nM), and simeprevir (*K_D_* = 812 nM) [17,18]. These molecules have displayed important interactions with residues of RBD and strong in vitro effects supporting the contention that blocking the viral entry by targeting the S protein is a successful strategy. In this context, Bojadzic et al. [19] have reported the development of dye-based PPI inhibitors, of which the compound DRI-C91005 (IC_50_ = 0.16 µM) displayed a potent effect on the cell-free ELISA-type assay. Therefore, the search for compounds with structural similarity to DRI-C91005 could yield new potent inhibitors of the interaction of the S protein with the ACE2 host receptor.

In this study, Ligand-Based Virtual Screening (LBVS) using compound DRI-C91005 was applied as a starting point to identify similar molecules with the potential to block the PPI by targeting the RBD region of the S protein, and finally, the best candidates were confirmed via an ELISA-based inhibition test as potential antiviral agents that prevent SARS-CoV-2 host cell entry.

## 2. Materials and Methods

### 2.1. Ligand-Based Virtual Screening (LBVS)

**Figure 2:**
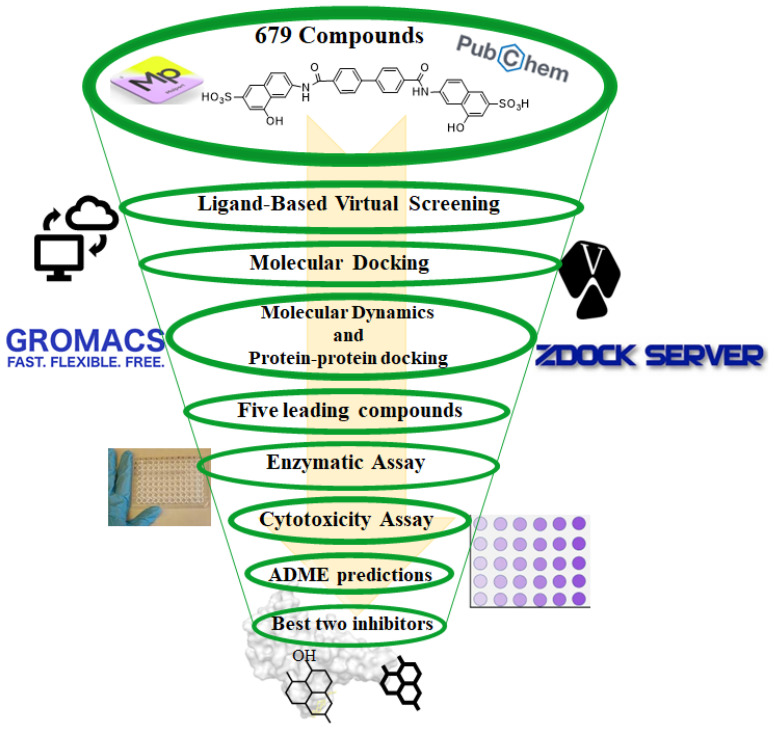
Workflow used in this study to identify inhibitors of the interaction of the SARS-CoV-2 spike protein and ACE2.

The study started with the LBVS carried out using the Mol-Port and PubChem databases using the substructure keys-based fingerprints (PubChem fingerprint) for similarity searching (Tanimoto coefficient cut-off = 0.5) with the compound DRI-C91005. The retrieved data were inspected to eliminate duplicates. The ligand minimization and addition of polar hydrogens was performed with OpenBabel 3.1.0 [20]. Figure 2 summarizes the workflow.

### 2.2. Molecular Docking

The crystallographic structure of the SARS-CoV-2 spike receptor-binding domain (RBD) bound with ACE2 (2.45 Å) was retrieved from the Protein Data Bank database (PDB code 6M0J). The USFC Chimera program was used to remove the co-crystallized human ACE2 chain, as well as other molecules [21]. The polar hydrogen addition and the side chain reparation was performed using the Chimera «DockPrep» command. The protonation state of the protein is only for the histidine residue considering a physiological pH. The Gasteiger charges addition and the PDBQT file conversion was performed with MGLTools 1.5.6 [22].

Molecular docking was performed using AutoDock Vina 1.1.2 [23]. The receptor (RBD) in PDBQT format was used to run Vina with a box size parameter of 19, 38, and 19 Å (X, Y, and Z, respectively) and was placed at the residues that actively participate in the SARS-CoV-2 spike–ACE2 binding Lys417, Gly446, Tyr449, Tyr453, Leu455, Phe456, Ala475, Phe486, Asn487, Tyr489, Gln493, G496, Gln498, Thr500, Asn501, Gly502, and Tyr505 (X = −36.496, Y = 28.684, and Z = 5.542). The other docking parameters were kept by default. Protein–Ligand Interaction Profiler (PLIP) was used to determine the non-covalent interactions between the ligand–receptor complex [24,25]. Once docking was completed, the best 20 ligands from each database were selected considering their score. In the next filter, the interactions with the residues of interest of all docking poses were analyzed, and the affinity of the pose with the best interactions was reported.

### 2.3. Protein–Protein Docking

The ZDOCK 3.0.2 server was used for the protein–protein docking studies [26]. According to the software instructions, the ACE2 receptor was considered as protein 1 (stationary), and the RBD with the corresponding inhibitor (complex from previous molecular docking) was called protein 2 and was treated as flexible. In addition, the original protein–protein complex (PDB ID: 6M0J) was run to predict the correct pose in the absence of ligands. Lastly, the S (RBD) and human ACE2 in the presence of inhibitors were evaluated to examine disruptions in the PPI. Specific residues of RBD (Lys417, Tyr449, Tyr453, Ala475, Asn487, Tyr489, Gly496, Thr500, Asn501, Gly502, and Tyr505) and ACE2 (Gln24, Thr27, Phe28, Asp30, Lys31, His34, Glu35, Glu37, Asp38, Tyr41, Gln42, Leu79, Met82, Tyr83, Asn330, Lys353, Gly354, Asp355, Arg357, and Arg393) were selected as flexible for the binding site during the docking run. The PDBsum platform was employed to analyze the PPI of the docking [27].

### 2.4. Molecular Dynamic Simulations

GROMACS version 2018.4 software was used to perform the molecular dynamic (MD) simulations at 200 ns at a 300 K temperature. The topology of each compound was generated with the ACPYPE Antechamber module using the General Amber Force Field [28]. The system was solvated adding water molecules in a dodecahedron box with a 10 Å minimum distance from the wall, using the TIP3P water model. Thereafter, the system was neutralized by adding Na^+^/Cl^−^ ions and energy-minimized using the steepest descent algorithm (50,000 times). The equilibrium steps were conducted at 300 K in two steps: (1) the ligand was simulated under NVT conditions (constant number of particles, volume, and temperature) using a V-rescale thermostat considering a time constant (tau_t) of 0.1 ps obtaining velocities according to the Maxwell–Boltzmann distribution; (2) the ligand was simulated under NPT conditions (constant number of particles, pressure at 1 atm, and temperature) utilizing a V-rescale thermostat and a Berendsen barostat with time constants (tau_t and tau_p) of 0.1 and 2.0 ps, respectively. Each step was achieved at 100 ps. The root mean square deviation (RMSD) and root mean square fluctuation (RMSF) calculations, using the GROMACS software tools v2018.4, were used to determinate the stability of each complex [29].

### 2.5. Biological Evaluation

#### 2.5.1. Enzymatic Inhibition Assay

The SARS-CoV-2 S1 Protein-ACE2 Binding Inhibitor Screening Kit (Cat #ab283370) was used to evaluate the compounds. Briefly, the control and the selected compounds were dissolved in dimethyl sulfoxide (DMSO, less than 1%) at 5 mM and stored at −80 °C until use. The plate with the pre-coated S1 protein was washed three times with 250 µL/well of 1X wash buffer (137 mM NaCl, 2.7 mM KCl, 10 mM Na₂HPO₄, 2 mM KH₂PO₄; Tween 0.05 % *v*/*v*; pH 7.4). Then, 50 µL of each compound at five concentrations prepared based on two-fold serial dilutions (50 to 3.125 µM) were added in duplicate into designated wells. The plate was protected from light and incubated at room temperature (rt) for 30 min, with gently shaking. Then, 50 µL of diluted biotinylated Human ACE2 was added to each well, and the plate was covered with a plate sealer and incubated at rt for 2 h shaking gently and protected from light. All reagents were aspirated, and each well was washed three times with 1X wash buffer. After the last wash, the plate was dried on absorbent filter paper. Immediately, 100 µL of Streptavidin-HRP (1:500) was added to each well and incubated for 1 h under the same conditions. Subsequently, the plate was washed as previously described, and TMB substrate (100 µL) was added to all wells; the color development was monitored for 2–20 min at rt. The reaction was stopped by adding 100 µL of Stop Solution to each well, and the absorbance at 450 nm was measured on a UV/Vis spectrophotometer (BioTek Epoch^®^, BioTek Instruments, Inc., Winooski, VT, USA). The relative inhibition was calculated using the following formula:

Relative inhibition (%) = (OD[Binding] − OD [S])/(OD[Binding]) × 100

where OD[Binding] is the optical density of the control without the inhibitor and OD[S] is the optical density of the sample compounds. The half-maximal inhibitory concentration (IC_50_) was determined using the GraphPad 6 statistical tool.

#### 2.5.2. Cytotoxicity

The mouse macrophage cell line J774.2 (ATCC^®^ TIB-67) was used for the evaluation of the in vitro cytotoxicity. Cells were cultured in RPMI medium supplemented with 10% SFB, 100 U µg/mL penicillin–100 mg/mL streptomycin, and glutamine (2 mM) at 37 °C and in an atmosphere of 5% CO_2_. The medium was changed at intervals of every 2 to 3 days. For the cytotoxicity assays, 1 × 10^6^ cells were incubated with different concentrations of the compounds (0.78 to 200 µM) at 37 °C for 48 h in an atmosphere of 5% CO_2_. Cells in the presence of the maximum concentration of DMSO (0.2%) were included as a negative control. The metabolic activity of the cells was determined using the MTT method. The percentage of cell viability was calculated, and the mean cytotoxic concentration (CC_50_) was determined via a Probit analysis. Three independent assays were performed in triplicate each. Finally, the selectivity index (SI) was calculated using the following formula CC_50_/IC_50_.

### 2.6. ADME-Tox Prediction

The Swiss-ADME (http://www.swissadme.ch/ accessed on 14 March 2024) online server was used to predict the pharmacokinetic properties (absorption, distribution, metabolism, and excretion) of the selected compounds [30]. For this purpose, each molecule was converted to a simplified molecular input line entry system (SMILES) format and entered as a single line in the corresponding field on the Swiss-ADME server. After a few seconds, the results were outputted, and the information was used to construct our tables.

## 3. Results

### 3.1. Ligand-Based Virtual Screening (LBVS) and Molecular Docking on RBD

**Figure 3:**
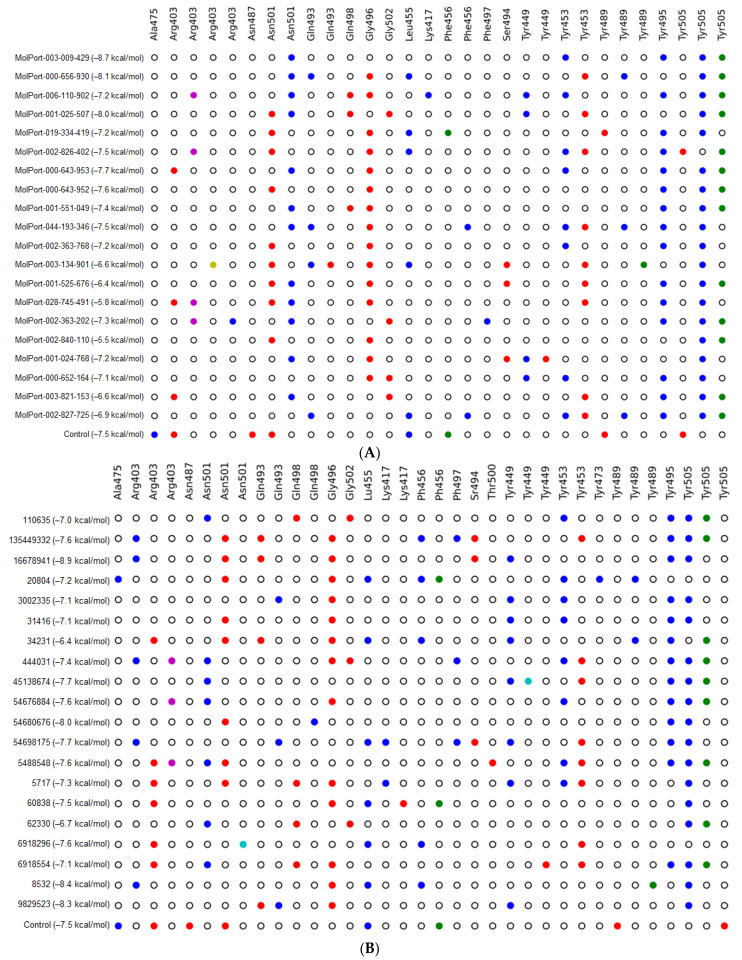
Interaction profile of the top 20 ligands with RBD residues. (**A**) Twenty ligands from the Mol-Port database with the best-predicted affinity; (**B**) twenty ligands from the PubChem database with the best-predicted affinity. Colors: white = no interaction, yellow = salt-bridge, blue = hydrophobic, sky-blue = halogen bond, red = hydrogen bond, green = π-stacking, purple= π-cation.

In this study, the compound DRI-C91005 was considered a scaffold to carry out an LBVS of the PubChem and Mol-Port databases (Tanimoto coefficient cut-off = 0.5). A total of 679 ligands were recovered from both databases, 99 compounds from Mol-Port (Supplementary Material, Table S1 Excel) and 580 from PubChem (Supplementary Material, Table S2 Excel). All ligands were evaluated based on molecular docking at the residues of the SARS-CoV-2 spike protein (RBD). These compounds displayed predicted binding affinity values ranked from –8.7 to –5.7 kcal/mol; meanwhile, the reference ligand (DRI-C91005) had a binding affinity of –7.5 kcal/mol. From this initial screening, the 20 ligands from each database with the best Vina score (Supplementary Material, Tables S3 and S4) were selected to analyze the nine docking poses to determine the protein–ligand interaction profile (Figure 3). In general, these compounds evidenced several hydrophobic interactions (Tyr505, Tyr945, and Asn501), hydrogen bonds (Gly496, Asn501, and Tyr453), and π-stacking (Tyr505).

**Figure 4:**
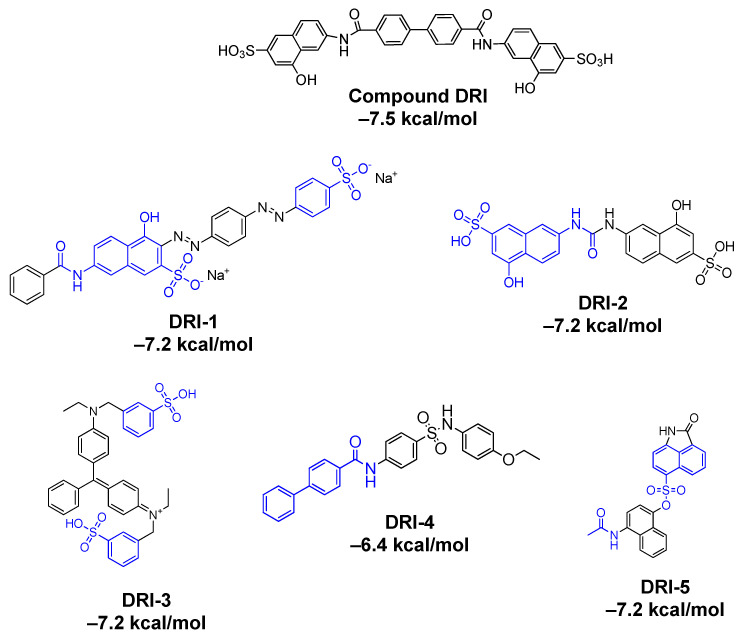
Chemical structures of compounds derived from DRI-based virtual screening. The structural similarities with the DRI compound are highlighted in blue. The affinity in kcal/mol is given according to the pose for interactions with residues of interest.

To maintain an adequate cost–benefit ratio and select the best inhibitors for the subsequent evaluation, an exhaustive revision was carried out of the availability, price, and amount (Tables S3 and S4) for the acquisition. The hit-five compounds (Figure 4) were chosen as MolPort-019-334-419 (DRI-1), MolPort-006-110-902 (DRI-2), 20804 (DRI-3), MolPort-001-525-676 (DRI-4), and MolPort-002-363-768 (DRI-5).

### 3.2. Protein–Protein Docking

**Figure 5:**
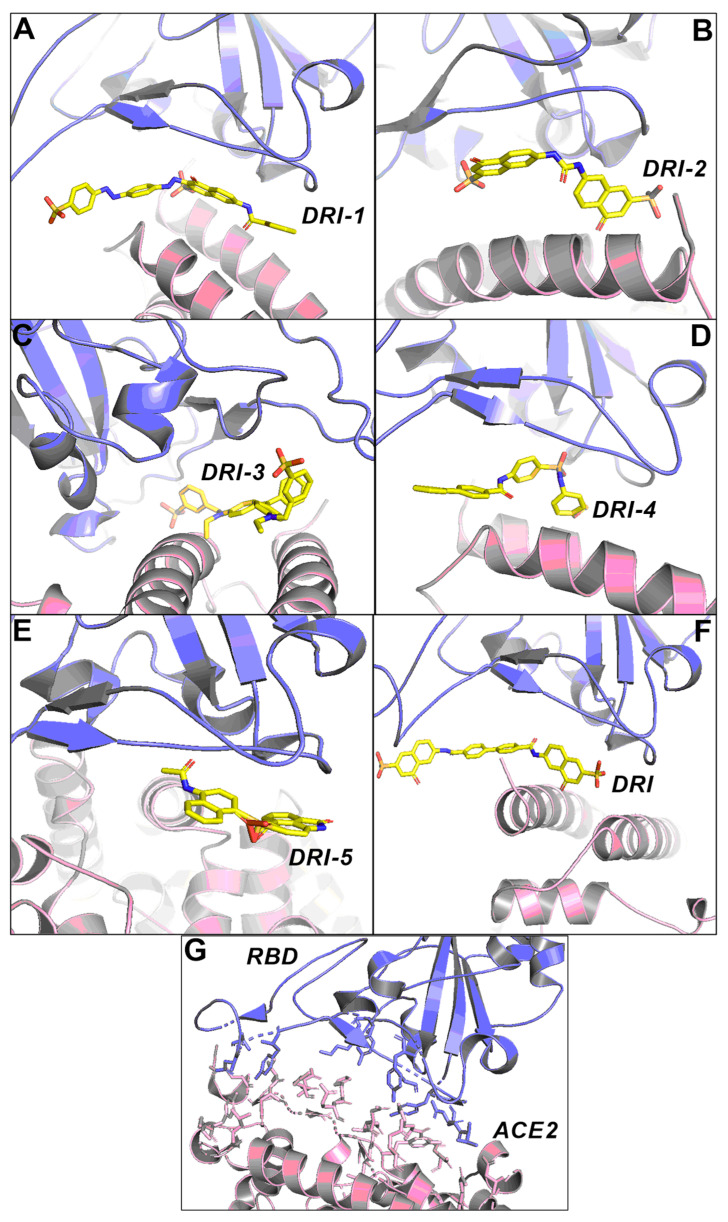
Putative docking pose of RBD (light blue) and human ACE2 (pink) in the presence of protein–protein inhibitors and absence of inhibitors. (**A**) putative binding mode of compound DRI-1 at the interface between RBD-ACE2 binding, (**B**) putative binding mode of compound DRI-2 at the interface between RBD-ACE2 binding, (**C**) putative binding mode of compound DRI-3 at the interface between RBD-ACE2 binding, (**D**) putative binding mode of compound DRI-4 at the interface between RBD-ACE2 binding, (**E**) putative binding mode of compound DRI-5 at the interface between RBD-ACE2 binding, (**F**) putative binding mode of compound DRI at the interface between RBD-ACE2 binding, and (**G**) interaction between RBD and ACE2 without inhibitors.

To infer the potential mechanism of blocking for the five selected compounds, protein-protein docking was carried out using the online server ZDOCK 3.0.2. First, a re-docking and root mean square deviation (RMSD) calculation was performed to validate the protocol using the complex RBD–ACE2 (PDB ID 6M0J). The RMSD value was of 0.98 Å (Figure S1), which is considered adequate. Then, each docking complex (RBD + inhibitor) was submitted to protein–protein docking under the same conditions. All the potential inhibitors disrupted the correct binding pose between the RBD and the human ACE2 (Figure 5).

The PDBsum platform allowed us to examine the protein–protein interactions. In general, the presence of inhibitors allowed the main interaction between RBD and ACE2 to be perturbed (Figures S2 and S3).

### 3.3. Molecular Dynamics

**Figure 6:**
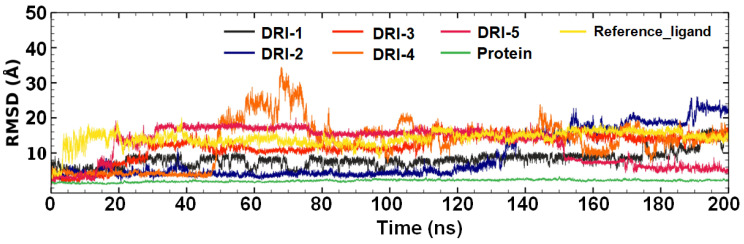
RMSD plot of DRI compounds (DRI-1 to DRI-5) in complex with RBD.

Molecular dynamic (MD) studies were carried out to identify and propose a potential mode of binding to the RBD of the SARS-CoV-2 spike protein. The five lead compounds were conducted at a total of 200 ns of simulation. First, the RBD-free protein was evaluated, which exhibited stability in terms of RMSD. Figure 6 shows the behavior of the protein with an RMSD value < 2 Å. To compare, the reference ligand DRI-C91005 in complex with RBD, it was simulated, and the stability was observed (RMSD = 14.1 Å) with minimal fluctuations (SD = 2.0 Å).

Compound DRI-1 in complex with RBD was evidenced to be stable in the first 130 ns, and the mean RMSD calculated was of 7.23 ± 1.5 Å. DRI-2 in complex with RBD displayed an acceptable stability in the first 135 ns. This complex showed a mean RMSD of 4.52 ± 1.3 Å from the total simulation. Complex RBD–DRI-3 had a mean RMSD of 12 ± 3.55 Å, a maximum RMSD value of 19.0 Å, and a minimum of 0.87 Å. The RBD–DRI-4 complex was stable in the first 45 ns (RMSD = 4.01 ± 0.4 Å), but the rest of the simulation showed abrupt changes; therefore, it was unstable. Finally, the compound DRI-5 in complex with RBD was analyzed, and the RMSD value of the total simulation was 12.4 ± 4.9 Å.

**Figure 7:**
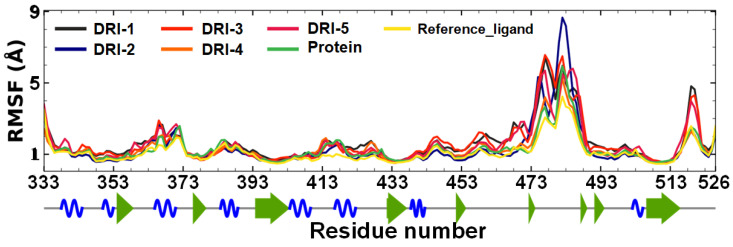
RMSF plot of DRI compounds (DRI-1 to DRI-5) in complex with RBD. The green arrow represents beta-sheets, the helix-alpha is shown as a spiral blue line, and the gray line corresponds to the loops.

Additionally, the root mean square fluctuation (RMSF) was examined during molecular dynamic simulations (Figure 7). The RMSF calculations for each ligand in complex with RBD showed minimal fluctuation patterns, except compound DRI-2, which had considerable fluctuation in a region corresponding to a loop of the RBD.

### 3.4. Biological Evaluations

#### 3.4.1. In Vitro Inhibition Assay

To validate the in silico predictions, an ELISA-based enzyme assay was performed for the selected compounds. All the compounds were screened at an initial concentration of 50 µM. The range of activity observed was from 69 to 99% of inhibition for the protein–protein interaction (Table 1). The positive control (a neutralizing antibody provided by the manufacturer) displayed 94.47% inhibition at 50 µM. The compound DRI-3 showed the most potent effect followed by the compound DRI-2 (Table 1).

#### 3.4.2. Cytotoxicity

In general, all the compounds evaluated displayed good values of cytotoxicity (CC_50_ ˃ 90 µM). Compound DRI-1 had a CC_50_ value of 94.5 µM, and the rest of the compounds had low cytotoxicity (>120 µM) in mouse macrophages. Additionally, the selectivity index (SI) was calculated for each compound. Compound DRI-1 showed the lowest SI value; meanwhile, compounds DRI-2 and DRI-3 had the highest values. The other compounds showed SI values close to 10.

### 3.5. ADME Predictions

Lastly, physicochemical properties and the absorption, distribution, metabolism, and excretion (ADME) parameters were calculated for all acquired compounds. The main descriptors obtained are shown in Table 2. In general, the compounds displayed acceptable drug-likeness properties.

## 4. Discussion

### 4.1. LBVS and Molecular Docking

Early studies for drug discovery against SARS-CoV-2 were based on in silico approaches because they are powerful tools for identifying active compounds. In this work, LBVS and SBVS were applied to investigate potential SARS-CoV-2 inhibitors. LBVS explores the information from active compounds that share similar chemical structures and physicochemical properties, which play a key role in binding at the target for biological activity. In this study, the compound DRI-C91005, which has been previously reported as a potent inhibitor [19], was considered as the initial hit to screen the PubChem and Mol-Port databases to identify new molecules analogues.

More than six hundred ligands were obtained as potential protein–protein inhibitors. All the ligands recovered were evaluated for molecular docking using AutoDock Vina 1.1.2 since it is accurate in predicting binding poses compared to others [31]. Then, the ligands were filtered by the Vina score as the first criterion. In this case, the range of the score was –8.7 kcal/mol to –5.7 kcal/mol (Supplementary Material, Tables S1 and S2); meanwhile, the reference ligand DRI-C91005 had a score of –7.5 kcal/mol. To obtain deep insight into the potential protein–protein inhibitors, only the twenty ligands with the best predicted affinity (–8.7 to –7.1 kcal/mol) were selected to further review the interactions with residues of interest. The main interactions observed were residues Lys417, Tyr449, Tyr453, Ala475, Asn487, Tyr489, Gly496, Thr500, Asn501, Gly502, and Tyr505, which is similar to those reported by some authors [32]. Prominent hydrogen bonds were detected for Gly496, whereas hydrophobic bonds were detected for Asn501, with π-stacking for Tyr505, which suggests that these interactions were essential for stabilizing the ligand–protein complex for these 20 compounds.

The hit compounds selected as potential inhibitors after, to be filtered by the best docking score, interactions with residues of interest, and availability to acquire commercially, were DRI-1, DRI-2, DRI-3, DRI-4, and DRI-5. Compound DRI-1, a dye derivative (Direct red 81) has been reported to be bioactive against malaria targeting the triosephosphate isomerase [33]. Compounds DRI-2 and DRI-4 have no reports of biological activity on any viral protein. Therefore, this is the first report related to protein inhibition. Compound DRI-3 (guinea green B, a dye for silk or wool and biological stains) at concentrations of 0.03, 0.3, and 3% has been shown to cause initial growth depression in rats, as well as the appearance of malignant tumors [34]. However, there are no more recent reports related to clinical studies. In the case of the compound DRI-5, in 2022, through SBVS studies, this molecule was proposed as a potential agent to treat COVID-19 targeting SARS-CoV-2 RNA-dependent RNA polymerase (RdRp) [35]. Despite this, in vitro validation still is not demonstrated.

### 4.2. Protein–Protein Docking

The ZDOCK (v3.0.2) online server was used for the protein–protein docking protocol of the S (RBD)–ACE2 complex in the presence and absence of inhibitors. ZDOCK v3.0.2 has an algorithm that explores all possible binding modes between the two proteins, using an energy-based scoring function to evaluate each pose [36]. A total of five poses for each complex were selected for analysis with the PBDsum server, and they were compared with the reference complex (PDB 6M0J) to investigate the differences in the residues of the protein–protein interaction. The five DRI-derived compounds disrupted the interaction between RBD and the ACE2 receptor. Based on the above, the presence of these compounds between the RBD and ACE2 causes a loss of interactions between RBD residues and ACE2.

### 4.3. Molecular Dynamics (MDs)

MD studies were conducted to analyze the stability of the RBD in complex with each inhibitor and identify a possible binding mode. The apo-protein (RBD) and the top five compounds were simulated at 200 ns. All MD runs were used to calculate the RMSD and RMSF. The RBD showed acceptable stability throughout the MD (RMSD < 2 Å). However, each PPI inhibitor and the reference ligand demonstrated stability at different times of the simulation. Hence, the stability of the complex is due to a different position from those observed in the initial docking pose. Hence, the stability of the complex is due to a different pose from those observed in the initial docking result. Therefore, we encourage the use of MD studies to gain insights into the protein–ligand interaction to propose a potential mechanism of inhibition of the SARS-CoV-2 spike protein targeting residues of the RBD. Additionally, calculations of RMSF showed very similar fluctuations for the free protein and the RBD in complex with compounds DRI.

### 4.4. Biological Activity

In general, the five compounds showed a concentration-dependent behavior in the enzymatic assays. To investigate the effect of different functional groups, a structure–activity relationship (SAR) analysis was carried out. The main groups identified in the compound DRI-C91005 were amide, hydroxyl, and sulfonic acid. The compounds DRI-1, DRI-2, and DRI-3 showed a sulfonic acid-free attached at the aromatic ring (phenyl or naphthyl), while the compound DRI-4 had a sulfonamide framework, and the compound DRI-5 possessed a sulfonate ester linker. These characteristics proved to be important for the formation of an interaction profile with residues of the RDB, which could result in its biological activity. Taken together, these findings showed that sulfonic acid is critical to retain the inhibition of the interaction between the S (RBD) protein and the ACE2 receptor. Likewise, the presence of bulky groups, such as biphenyl and naphthyl, are keys to efficiently binding the RBD of the spike protein, which can be explained by the hydrophobic nature of some pockets of the RBD that have been previously described [37].

Finally, cytotoxicity studies in mouse macrophages demonstrated that the compounds are not harmful up to concentrations of 100 µM, except for the compound DRI-1. Additionally, the selectivity index (SI) was calculated from the CC_50_ values and the inhibitory effect (IC_50_). Compounds exhibited acceptable SI values (>5), specifically compound DRI-3 (SI = 93.4) and compound DRI-2 (SI = 22.6) that reached the highest values even above the SI value recommended (≥10) by Indrayanto et al. [38]; therefore, they are promising molecules for future studies in other cell lines and studies with infected SARS-CoV-2 cells to demonstrate their therapeutic potential as antiviral agents.

### 4.5. ADME Predictions

The SwissADME server was used to predict some physicochemical properties and pharmacokinetic parameters of the evaluated compounds. The DRI-4 and DRI-5 derivatives presented a molecular weight < 500 g/mol; therefore, they are considered “small molecules”. The compounds DRI-1 and DRI-3, and the reference ligand DRI had a molecular weight greater than 500 g/mol. Only DRI-2 slightly exceeded the molecular weight. Three compounds (DRI-1, DRI-2, and DRI-3) had some properties beyond the established range, such as an MW > 500, rotatable bonds > 10, HBA > 10, HBD > 5, and TPSA > 140 Å. These predictions may influence the future therapeutic profile of these compounds. Therefore, structural optimization would be helpful to improve pharmacokinetics and advance to preclinical and clinical trials. In this sense, the incorporation of esters into the free sulfonic acid of lead compounds may provide sulfonate derivatives that improve the ADME profile. In addition, a violation of this rule, such as the molecular weight, is not a constraint to progress to preclinical and/or clinical trials if other characteristics are desirable [39,40].

All compounds showed a predicted low gastrointestinal absorption (except DRI-5) and were not capable of permeating the blood–brain barrier. Therefore, for further clinical assays, gastrointestinal absorption can be improved using substances, such as intestinal permeation enhancers (PEs) [41]. The compounds DRI-1 and DRI-4 were substrates of the P-glycoprotein. This protein uses ATP to function as a transmembrane pump for the unidirectional extracellular outflow of several substances, which could reduce the concentration of the compounds inside the cell and diminish their effect [42]. In addition, most CYP450 isoforms showed a variable probability of being inhibited by the compounds. Only compound DRI-4 was shown to block isoform CYP3A4, which is key in xenobiotic detoxification [43]; therefore, in vitro validation assays are required to establish an improvement in pharmacokinetics for this compound.

## 5. Conclusions

In this work, a combination of in silico and in vitro protocols helped to identify compounds capable of blocking the interaction between the spike protein and the ACE2 receptor. Compounds DRI-2 and DRI-3 had activity in the micromolar range (<10 µM) with minimal cytotoxicity in macrophages (CC_50_ > 200 µM). Based on the above, we consider that the compounds in this study are promising hits to determine whether they inhibit SARS-CoV-2 entry by targeting the spike protein (RBD), which is required for in vivo evaluation. Additionally, in drug discovery, the protein–ligand interaction provides better knowledge to develop inhibitors, and in this scenery, we consider that the crystallization of the spike in complex with compounds DRI-2 and DRI-3 would provide a more detailed understanding of the binding site and the molecular mechanism involved in the interaction of the protein–ligand, in comparison to those observed in molecular docking and MD studies; this could help in the design of potent antivirals acting on viral entry.

## Figures and Tables

**Table 1:** Percentage of inhibition, half maximal inhibitory concentration blocking the binding of SARS-CoV-2 spike RBD to human ACE2, and half maximal cytotoxic concentration against the macrophage cell line J774.2 of DRI-compound derivatives.

Compound	(%) Inhibition at 50 µM	IC_50_ (µM) ^a^	CC_50_ (µM) ^a^	SI
**DRI-1**	90.59 ± 0.54	13.04 ± 0.71	94.55 ± 0.96	7.25
**DRI-2**	90.76 ± 1.7	8.84 ± 4.3	˃200	22.62
**DRI-3**	99.65 ± 0.25	2.14 ± 0.97	˃200	93.45
**DRI-4**	76.41 ± 1.0	24.55 ± 2.5	˃200	8.14
**DRI-5**	69.90 ± 1.2	13.70 ± 0.12	126.25 ± 2.30	9.21

^a^ Values are the means of three experiments.

**Table 2:** ADME predictions of selected compounds.

Physicochemical Properties and Drug-Likeness									
Compound	MW (g/mol) ≤ 500	Rot. Bonds < 10	HBA < 10	HBD < 5	Log P < 5	Log S	TPSA (≤140 Å²)	Lipinski’s Rule	Veber’s Rule
**DRI-1**	675.60	9	12	2	-0.81	Poor	229.93	2 violations	1 violation
**DRI-2**	504.49	6	9	6	1.80	Moderate	207.09	3 violations	1 violation
**DRI-3**	669.83	11	6	2	4.65	Poor	131.75	2 violations	1 violation
**DRI-4**	472.56	9	4	2	4.69	Poor	92.88	0 violation	0 violation
**DRI-5**	432.45	5	5	2	3.61	Moderate	113.71	0 violation	0 violation
**DRI**	684.69	9	10	6	3.86	Poor	224.16	3 violations	1 violation
**Pharmacokinetics**									
**Compound**	**Blood-Brain Permeability**	**GI Absorption**	**P-Glycoproteinsubstrate**	**CYP1A2** **Inhibitor**	**CYP2C19 Inhibitor**	**CYP2C9 Inhibitor**	**CYP2D6 Inhibitor**	**CYP3A4** **Inhibitor**	**PAINS**
**DRI-1**	No	Low	Yes	No	No	No	No	No	1 alert
**DRI-2**	No	Low	No	No	No	No	No	No	0 alert
**DRI-3**	No	Low	No	No	No	Yes	No	No	1 alert
**DRI-4**	No	Low	Yes	No	Yes	Yes	Yes	Yes	0 alert
**DRI-5**	No	High	No	Yes	Yes	Yes	No	No	0 alert
**DRI**	No	Low	No	No	No	No	No	No	0 alert

MW: molecular weight; Rot. Bonds: rotatable bonds; HBA: hydrogen bond acceptor; HBD: hydrogen bond donor; Log P: Consensus Log P; Log S: water solubility; TPSA: topological polar surface area. PAINS: pan assay interference structures.

## Data Availability

Data are contained within the article and Supplementary Material.

## Supplementary Materials

The following supporting information can be downloaded at: https://www.mdpi.com/article/10.3390/pharmaceutics16050613/s1, Figure S1: Comparison of protein–protein complex between RBD and ACE2 (re-docking versus original). Figure S2: Interaction between RBD and ACE2 in the presence of inhibitors (PPI). Figure S3: Profile of interaction of protein–protein complex (PDB 6M0J). Table S1: List of the 99 ligands recovered from Mol-Port (Sheet-1_MolPort_Database) and evaluated based on molecular docking (Sheet-2_MolPort_Vina_Score). Table S2: List of the 580 ligands recovered from PubChem (Sheet-1_PubChem_Database) and evaluated based on molecular docking (Sheet-2_PubChem_Vina_Score). Table S3: List of top 20 ligands with best Vina score recovered from Mol-Port. Table S4: List of top 20 ligands with best Vina score recovered from PubChem.
