# Supplementary material for: Ligand- and Structure-Based Virtual Screening Identifies New Inhibitors of the Interaction of the SARS-CoV-2 Spike Protein with the ACE2 Host Receptor
Source: Pharmaceutics. 2024 May 1;16(5):613. doi: 10.3390/pharmaceutics16050613 (PMC11124852; doi:10.3390/pharmaceutics16050613)
Supplement: Supplementary file 1 [file pharmaceutics-16-00613-s001.zip › Supplementary Files.pdf]

Supplementary Materials

The supplementary material corresponds to the *in silico* studies.

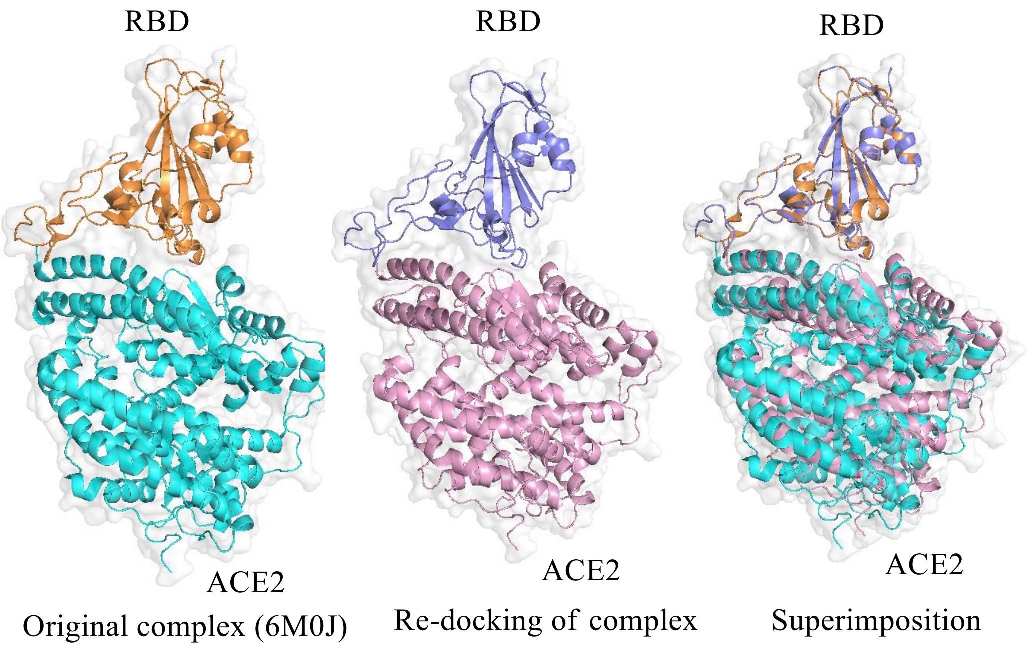

Figure S1. Comparison of protein-protein complex between RBD and ACE2 (re-docking versus original).

Table S3. List of top 20 ligands with best vina score recovered from Mol-Port.

Number	Mol-Port ID	Internal code	Vina score (kcal/mol)	Availability	Amount/Price
1	MolPort-003-009-429	DRI-C24041_MP_SIM_50_U_19_out.pdbqt	-8.7	In stock	10 mg/99.00 USD
2	MolPort-000-656-930	DRI-C24041_MP_SIM_50_U_14_out.pdbqt	-8.4	In stock	10 mg/275.00 USD
3	MolPort-006-110-902	DRI-C24041_MP_SIM_50_U_25_out.pdbqt	-8	In stock	1 g/6.00 USD
4	MolPort-001-025-507	DRI-C24041_MP_SIM_50_U_88_out.pdbqt	-8	In stock	10 mg/134.01 USD
5	MolPort-019-334-419	DRI-C24041_MP_SIM_50_U_5_corregido_out.pdbqt	-7.9	In stock	I g/10.00 USD
6	MolPort-002-826-402	DRI-C24041_MP_SIM_50_U_2_out.pdbqt	-7.8	In stock	10 mg/22.80 USD
7	MolPort-000-643-953	DRI-C24041_MP_SIM_50_U_69_out.pdbqt	-7.7	In stock	10 mg/399.00 USD
8	MolPort-000-643-952	DRI-C24041_MP_SIM_50_U_70_out.pdbqt	-7.6	In stock	5 mg/87.77 USD
9	MolPort-001-551-049	DRI-C24041_MP_SIM_50_U_73_out.pdbqt	-7.6	In stock	10 mg/399.00 USD
10	MolPort-044-193-346	DRI-C24041_MP_SIM_50_U_31_corregido_out.pdbqt	-7.5	In stock	5 mg/133.10 USD

11	MolPort-002-363-768	DRI-C24041_MP_SIM_50_U_80_out.pdbqt	-7.5	In stock	10 mg/22.80 USD
12	MolPort-003-134-901	DRI-C24041_MP_SIM_50_U_38_out.pdbqt	-7.3	In stock	10 mg/99.00 USD
13	MolPort-001-525-676	DRI-C24041_MP_SIM_50_U_47_out.pdbqt	-7.3	In stock	10 mg/22.80 USD
14	MolPort-028-745-491	DRI-C24041_MP_SIM_50_U_48_out.pdbqt	-7.3	Discontinued	-
15	MolPort-002-363-202	DRI-C24041_MP_SIM_50_U_65_out.pdbqt	-7.3	In stock	10 mg/22.80 USD
16	MolPort-002-840-110	DRI-C24041_MP_SIM_50_U_75_out.pdbqt	-7.3	In stock	10 mg/61.25 USD
17	MolPort-001-024-768	DRI-C24041_MP_SIM_50_U_10_out.pdbqt	-7.2	In stock	10 mg/61.25 USD
18	MolPort-000-652-164	DRI-C24041_MP_SIM_50_U_40_out.pdbqt	-7.2	In stock	10 mg/99.00 USD
19	MolPort-003-821-153	DRI-C24041_MP_SIM_50_U_8_out.pdbqt	-7.2	In stock	10 mg/22.80 USD
20	MolPort-002-827-725	DRI-C24041_MP_SIM_50_U_34_out.pdbqt	-7.1	In stock	10 mg/22.80 USD

Table S4. List of top 20 ligands with best vina score recovered from PubChem.

Number	PubChem ID	Internal code	Vina score (kcal/mol)	Availability	Amount/Price
1	16678941	DRI-C24041_PC_SIM50_380_out.pdbqt	-8.9	In stock	10 mg/379.50 USD
2	8532	DRI-C24041_PC_SIM50_486_out.pdbqt	-8.4	In stock	10 mg/399.00 USD
3	9829523	DRI-C24041_PC_SIM50_201_out.pdbqt	-8.3	In stock	10 mg/568.00 USD
4	60838	DRI-C24041_PC_SIM50_79_out.pdbqt	-8.3	In stock	25 mg/80.00 USD
5	54680676	DRI-C24041_PC_SIM50_4_out.pdbqt	-8	In stock	50 mg/562.00 USD
6	54676884	DRI-C24041_PC_SIM50_179_out.pdbqt	-7.9	In stock	25 mg/134.70 USD
7	34231	DRI-C24041_PC_SIM50_271_out.pdbqt	-7.8	In stock	250 mg/12.82.00 USD
8	110635	DRI-C24041_PC_SIM50_231_out.pdbqt	-7.7	In stock	50 mg/266.00 USD
9	54698175	DRI-C24041_PC_SIM50_367_out.pdbqt	-7.7	not available for purchase	-
10	45138674	DRI-C24041_PC_SIM50_374_out.pdbqt	-7.7	In stock	1 mg/104.50 USD
11	62330	DRI-C24041_PC_SIM50_433_out.pdbqt	-7.7	In stock	10 mg/399.00 USD
12	31416	DRI-C24041_PC_SIM50_452_out.pdbqt	-7.7	In stock	1 mg/80.45 USD
13	135449332	DRI-C24041_PC_SIM50_173_out.pdbqt	-7.6	In stock	10 mg/167.20 USD
14	6918296	DRI-C24041_PC_SIM50_203_out.pdbqt	-7.6	In stock	10 mg/185.90 USD
15	5488548	DRI-C24041_PC_SIM50_205_out.pdbqt	-7.6	In stock	10 mg/298.00 USD
16	444031	DRI-C24041_PC_SIM50_410_out.pdbqt	-7.6	In stock	100 mg/300.00 USD
17	6918554	DRI-C24041_PC_SIM50_202_out.pdbqt	-7.5	In stock	10 mg/30.00 USD
18	3002335	DRI-C24041_PC_SIM50_217_out.pdbqt	-7.5	In stock	1 g/332.00 USD
19	20804	DRI-C24041_PC_SIM50_280_out.pdbqt	-7.5	In stock	5 g/30.00 USD
20	5717	DRI-C24041_PC_SIM50_314_out.pdbqt	-7.5	In stock	10 mg/76.00 USD
21	163233165	DRI-C24041_Control_out.pdbqt	-7.5	-	-

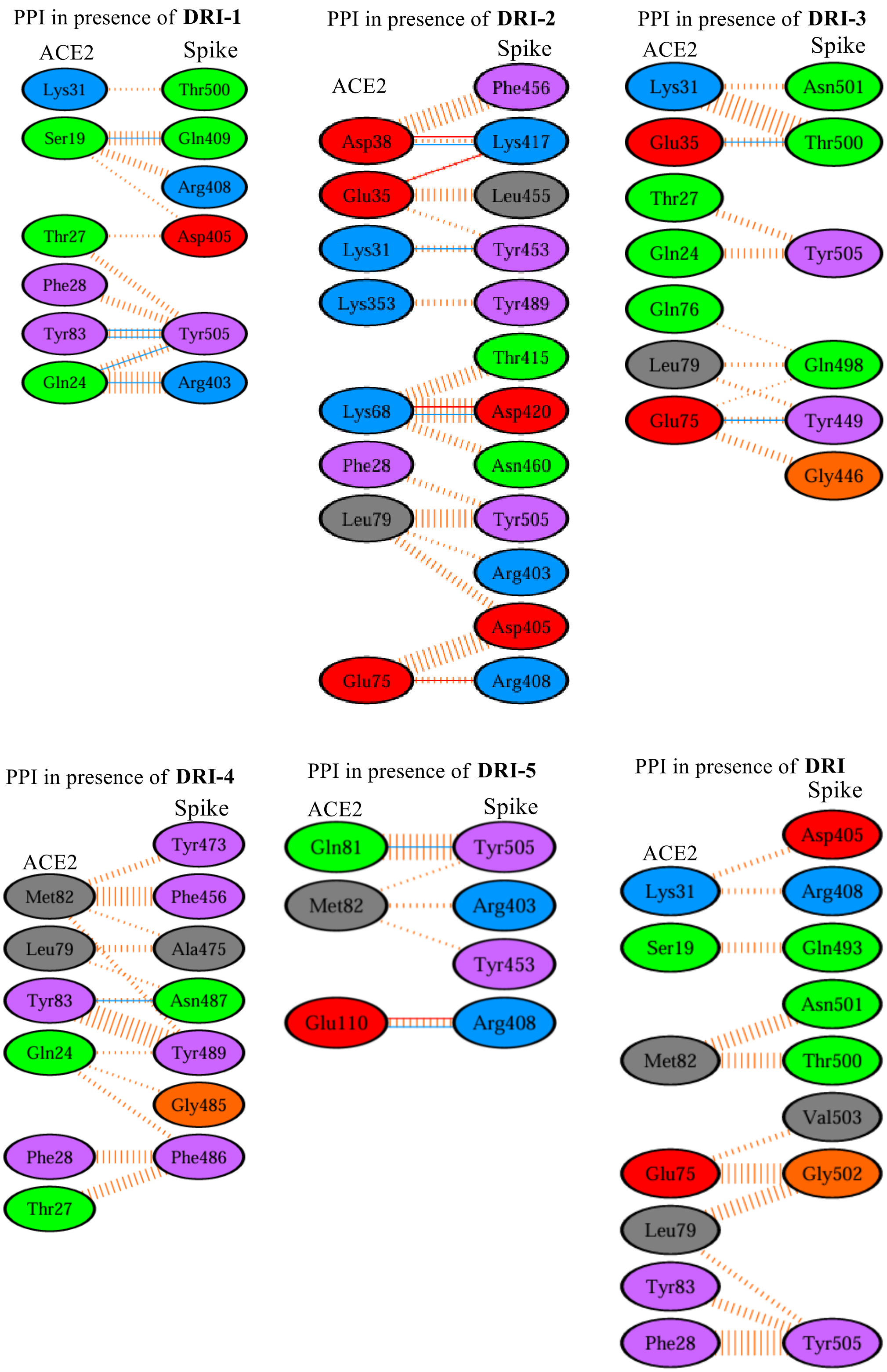
PPI in presence of DRI-1
ACE2
Spike
Lys31
Thr500
Ser19
Gln409
Arg408
Thr27
Asp405
Phe28
Tyr83
Tyr505
Gln24
Arg403
PPI in presence of DRI-2
Spike
ACE2
Phe456
Asp38
Lys417
Glu35
Leu455
Lys31
Tyr453
Lys353
Tyr489
Thr415
Lys68
Asp420
Phe28
Asn460
Leu79
Tyr505
Arg403
Asp405
Glu75
Arg408
PPI in presence of DRI-3
ACE2
Spike
Lys31
Asn501
Glu35
Thr500
Thr27
Gln24
Tyr505
Gln76
Leu79
Gln498
Glu75
Tyr449
Gly446
PPI in presence of DRI-4
Spike
ACE2
Tyr473
Met82
Phe456
Leu79
Ala475
Tyr83
Asn487
Gln24
Tyr489
Gly485
Phe28
Phe486
Thr27
PPI in presence of DRI-5
ACE2
Spike
Gln81
Tyr505
Met82
Arg403
Tyr453
Glu110
Arg408
PPI in presence of DRI
Spike
ACE2
Asp405
Lys31
Arg408
Ser19
Gln493
Asn501
Met82
Thr500
Val503
Glu75
Gly502
Leu79
Tyr83
Phe28
Tyr505

Figure S2. Interaction between RBD and ACE2 in presence of potential protein-protein inhibitors (PPI). Salt bridges are shown as red lines, yellow lines represent disulfide bonds, Hydrogen bonds are illustrated as sky-blue color and orange lines mean non-bonded contacts. Residue colours: Positive (His, Lys, and Arg); negative (Asp, Glu); Ser, Thr, Asn, Gln = neutral; Ala, Val, Leu, Ile, Met = aliphatic; Phe, Tyr, Trp = aromatic; P, G = Pro, Gly; C = cysteine.

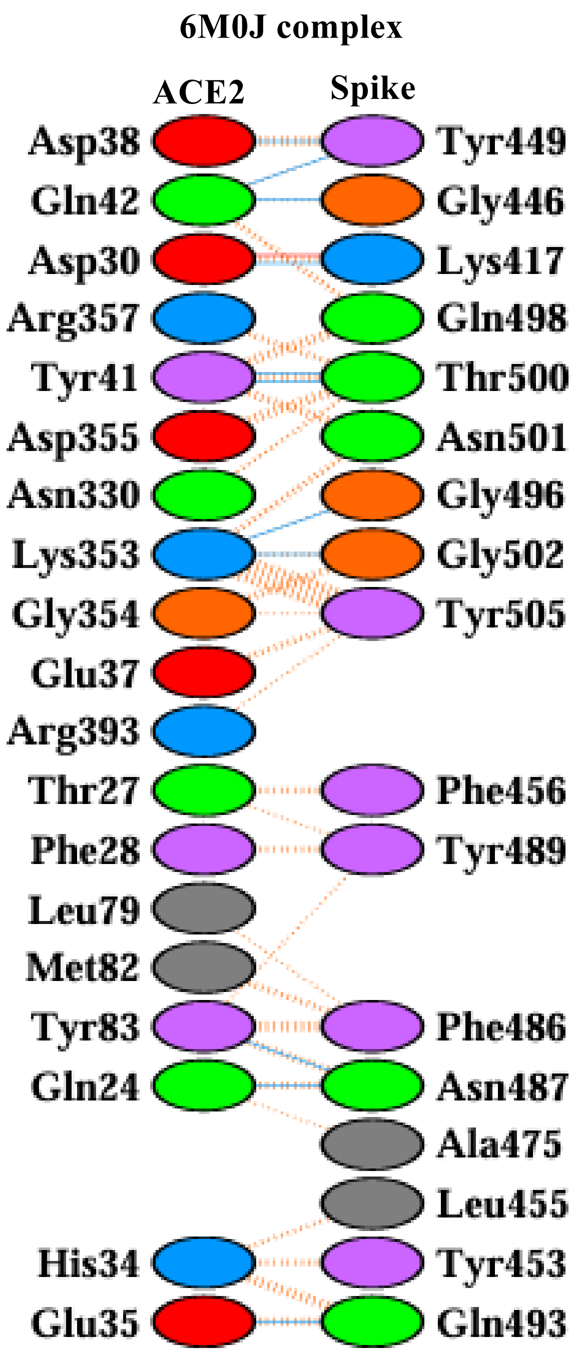

Figure S3. Protein-protein interaction profile of the original crystal (PDB ID: 6M0J). Salt bridges are shown as red lines, yellow lines represent disulfide bonds, Hydrogen bonds are illustrated as sky-blue color and orange lines mean non-bonded contacts. Residue colours:

Positive (His, Lys, and Arg); negative (Asp, Glu); Ser, Thr, Asn, Gln = neutral; Ala, Val, Leu, Ile, Met = aliphatic; Phe, Tyr, Trp = aromatic; P, G = Pro, Gly; C = cysteine.
